# Renal replacement therapy in ADPKD patients: a 25-year survey based on the Catalan registry

**DOI:** 10.1186/1471-2369-14-186

**Published:** 2013-09-05

**Authors:** Víctor Martínez, Jordi Comas, Emma Arcos, Joan Manel Díaz, Salomé Muray, Juan Cabezuelo, José Ballarín, Elisabet Ars, Roser Torra

**Affiliations:** 1 Nephrology Department, Hospital Reina Sofia, Murcia, Spain; 2 Catalan Renal Registry, Catalan Transplant Organization, Health Department, Generalitat of Catalonia, Barcelona, Spain; 3 Nephrology Department, Fundacio Puigvert, IIB Sant Pau, Univeristat Autónoma de Barcelona, Barcelona, Spain; 4 Molecular Biology Laboratory, Fundacio Puigvert, IIB Sant Pau, Univeristat Autónoma de Barcelona, Barcelona, Spain; 5 Inherited Renal Diseases, Nephrology Department, Fundacio Puigvert, IIB Sant Pau, Universitat Autónoma de Barcelona, Barcelona, Spain

**Keywords:** Autosomal dominant polycystic kidney disease, Renal replacement therapy, Survival, Co-morbidities, ADPKD

## Abstract

**Background:**

Some 7-10% of patients on replacement renal therapy (RRT) are receiving it because of autosomal dominant polycystic kidney disease (ADPKD). The age at initiation of RRT is expected to increase over time.

**Methods:**

Clinical data of 1,586 patients (7.9%) with ADPKD and 18,447 (92.1%) patients with other nephropathies were analysed from 1984 through 2009 (1984–1991, 1992–1999 and 2000–2009).

**Results:**

The age at initiation of RRT remained stable over the three periods in the ADPKD group (56.7 ± 10.9 (mean ± SD) vs 57.5 ± 12.1 vs 57.8 ± 13.3 years), whereas it increased significantly in the non-ADPKD group (from 54.8 ± 16.8 to 63.9 ± 16.3 years, p < 0.001). The ratio of males to females was higher for non-ADPKD than for ADPKD patients (1.6–1.8 vs 1.1–1.2). The prevalence of diabetes was significantly lower in the ADPKD group (6.76% vs 11.89%, p < 0.001), as were most of the co-morbidities studied, with the exception of hypertension. The survival rate of the ADPKD patients on RRT was higher than that of the non-ADPKD patients (p < 0.001).

**Conclusions:**

Over time neither changes in age nor alterations in male to female ratio have occurred among ADPKD patients who have started RRT, probably because of the impact of unmodifiable genetic factors in the absence of a specific treatment.

## Background

Autosomal dominant polycystic kidney disease (ADPKD) is an inherited disease caused by mutations in two genes, *PKD1* and *PKD2*, which encode for polycystin 1 and polycystin 2, respectively: mutations in *PKD1* are present in 85% of cases, and mutations in *PKD2* in the remaining 15% [1-3]. The abnormalities in these proteins cause the appearance and growth of kidney cysts that gradually impair renal function [1]. ADPKD is the cause of chronic kidney disease (CKD) in 5% of patients undergoing renal replacement therapy (RRT) in the USA [4], a rate that rises to 7.9% in our geographic area [5]. In 75-82% of patients it is additionally associated with hypertension, which often appears before CKD development [6], and with other systemic disturbances such as liver cysts, pancreatic cysts, brain aneurysms or colonic diverticula.

Progression of CKD in ADPKD basically depends on a combination of genetic and environmental factors, with hypertension as a significant prognostic factor. CKD is more severe and appears earlier in patients with *PKD1* gene mutations than in those with *PKD2* mutations (mean age at RRT initiation: 53 and 69 years, respectively) [3,7]. Also, the prognosis is worse for those harbouring a truncating *PKD1* mutation compared with those with missense mutations [8]. Various studies have shown that controlling hypertension slows down CKD development in patients with ADPKD [9]. A recent trial, however, has indicated that reduced glomerular filtration is a function of older age, and that there are no differences between hypertensive and normotensive patients [10]; hypertensionis present in nearly 90% of ADPKD patients once they have renal failure.

CKD is associated with a high risk of cardiovascular disease: cardiovascular mortality among dialysis patients is 10–30 times greater than among the general population [11]. This fact is explained by the higher prevalence of classic cardiovascular risk factors in such patients: hypertension, diabetes, dyslipidaemia and smoking [12]. Diabetic nephropathy and vascular nephropathy are the primary reasons for initiation of dialysis, specially among the North American population [4,5]. Moreover, a slowdown in CKD progression has been verified when cardiovascular risk factors are controlled [13]. However, a lower prevalence of these risk factors has been described in patients with ADPKD, with the sole exception of hypertension [14], which is present in nearly 90% of ADPKD patients once they have renal failure.

The objective of this observational study is to analyse the epidemiological, clinical and survival changes that occur over time in ADPKD patients who have started RRT, as compared with non-ADPKD patients on RRT during the last 25 years.

## Methods

### Study design

The data analysed came from the Catalan Renal Patients Registry, created in 1984 with the aim of assisting the Renal Failure Care Programme in planning its resources. This is a mandatory reporting registry for all patients in Catalonia, a Spanish autonomous community with over 7 million inhabitants. This registry collects socio-demographic and clinical data of all patients who start RRT, including haemodialysis, peritoneal dialysis and renal transplantation and excluding transient patients. The quality of the information is ensured by means of mechanisms of validation and processing of the periodically reported cases, and the performance of a yearly follow-up of all active cases as of 31 December.

Patients who started RRT between 1984 and 2009 were considered and divided into two groups: patients on RRT because of ADPKD [code 41 in the ERA-EDTA classification of Primary Renal Disease (PRD)], and the remaining patients with other reasons for initiation of RRT (remaining ERA-EDTA PRD codes), called the non-ADPKD group. The diagnosis of ADPKD was specified by the clinician at the time of reporting to the Registry, based on the patient’s family history and on ultrasound criteria compatible with the disease. Patients actively on dialysis at 31 December 2009 were also considered when describing the current situation of patients.

### Clinical and demographic data

The study follow-up time was divided into three periods of RRT initiation: 1984–1991, 1992–1999 and 2000–2009.

The variables analysed were gender and age, both upon RRT initiation and at the end of the RRT study, and the presence of associated diseases in the 1992–2009 cases. These associated diseases included: hypertension (defined as blood pressure over 140/90), diabetes mellitus, ischaemic or non-ischaemic heart disease, arrhythmia, cerebrovascular disease, peripheral vascular disease, malignancies, chronic respiratory disease, tuberculosis, chronic liver disease, gastroduodenal disorders, bowel disease and joint disease. These co-morbidities were analysed from 2000 to 2009 because from 1992 to 1999 it was not compulsory to notify them and therefore data were not complete.

The first vascular access (temporary catheter, tunnelled catheter, arteriovenous fistula or prosthetic graft) was also analysed in patients who started haemodialysis between 1997 and 2009, divided into two periods: 1997–2003 and 2004–2009.

In patients actively on dialysis as of 31 December 2009, we studied: percentage of patients with hepatitis C virus, percentage of patients who received erythropoietin, and C-reactive protein (CRP) levels in mg/l (CRP >10 mg/l or <10 mg/l), according to the standard method.

In patients who had received a kidney transplant we analysed the type of donor (living or cadaveric), the time elapsed since start of dialysis to transplant surgery, and the age at the time of transplantation.

Regarding those patients who died while on RRT, we analysed their age at the time of death and the causes of death. We classified the causes of death as follows: cardiac, vascular, infectious, cancer, hepatic, social, other and unknown. Survival rates of patients with and without ADPKD during the first 3 years after initiation of RRT, and also patient and renal graft survival from the time of transplantation, when applicable, were also considered.

### Statistical analysis

Software STATA11 was used for statistical analysis. Chi-square test was applied to analyse qualitative variables, and Student’s t-test for the comparison of means of the quantitative variable. The log-rank test was used to verify the survival rates. Statistical significance was set at p < 0.05.

### Limitations

Only the information contained in the fields of the mandatory reporting registry of the Catalan Renal Patients Registry has been used in this observational study. Other interesting information for the purpose of this study, but not included in these forms, was not available. For example, data relating to the period prior to the onset of end-stage renal disease (ESRD) were not available as the study is based on a registry of ESRD.

## Results

A total of 20,033 patients in whom RRT had been initiated were registered between 1984 and 2009: 1,586 of them (7.9%) had ADPKD and 18,447 (92.1%) had other nephropathies.

**Figure 1:**
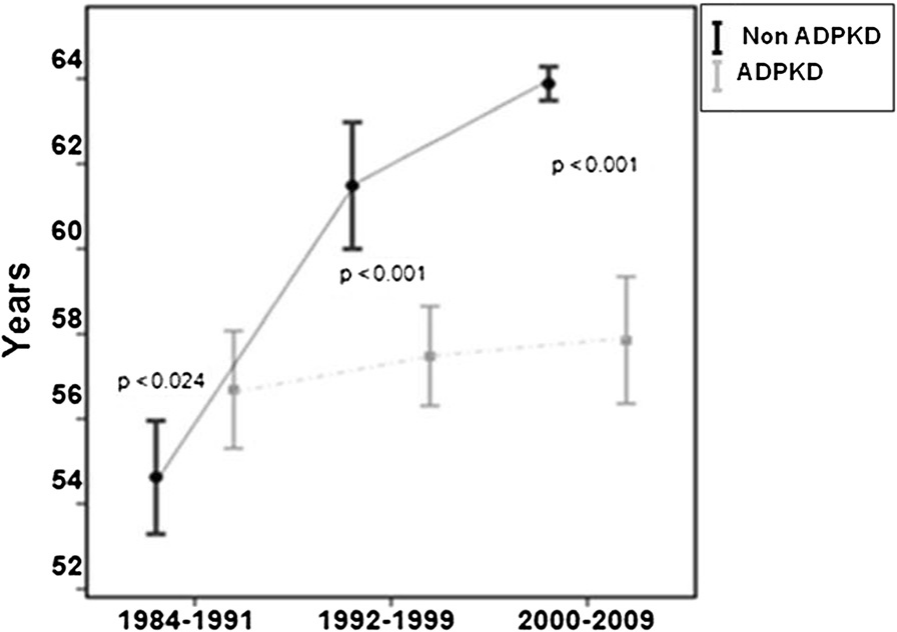
Mean age at initiation of RRT for ADPKD and non-ADPKD patients for the three different periods of the study (p < 0.01).

As shown by Table 1 and Figure 1, age at initiation of RRT in the ADPKD group was significantly lower than in the remaining patients, for both males and females (p < 0.001). Among ADPKD patients, the mean age over the three periods investigated was 56.7 ± 10.9, 57.5 ± 12.1 and 57.8 ± 13.3 years, respectively, with no significant differences (p = 0.37). Among non-ADPKD patients, however, the mean age increased significantly (p < 0.001) as follows: from 54.8 ± 16.8 years in the first period to 61.7 ± 16.2 years in the second period to 63.9 ± 16.3 years in the third period.

**Table 1:** Clinical features of ADPKD and non-ADPKD patients on RRT

	**ADPKD (n: 1586)**	**Non ADPKD (n: 18,447)**	***p***
Age (years) at initiation of RRT in men	57.2 ± 12.7	60.9 ± 16.6	<0.001
Age (years) at initiation of RRT in women	57.8 ± 12.0	62.5 ± 17.0	<0.001
First choice of RRT (%)	HD 91.1%	HD 89.4%	<0.01
	TX 2.5%	TX 8.5%	
	PD 6.4%	PD 2.0%	
RRT strategy at 31.12.2009 (%)^1^	HD 31.8%	HD 46.9%	<0.001
	TX 65.4%	TX 49.3%	
	PD 2.8%	PD 3.8%	
Time from initiation of RRT to TX (years)^2^	2.9 (2.7-3.1)	3.1 (3.0-3.1)	p = 0.09
Age at TX (years)^2^	53.5 (52.9-54.0)	44.6 (44.2-44.9)	<0.001
Treated with ESA (%)^1^	77.9%	91.9%	<0.001
HCV (%)^1^	5.1%	10.0%	<0.001
CRP (<10 mg/l)^1^	71.7%	66.9%	0.124

*ADPKD* Autosomal dominant polycystic kidney disease, *RRT* renal replacement therapy, *HD* haemodialysis, *TX* transplantation, *PD* peritoneal dialysis, *ESA* erythropoiesis stimulating agents, *HCV* hepatitis C virus, *CRP* C-reactive protein.

^1^Patients actively on RRT at 31 December 2009, ^2^95% confidence intervals.

**Figure 2:**
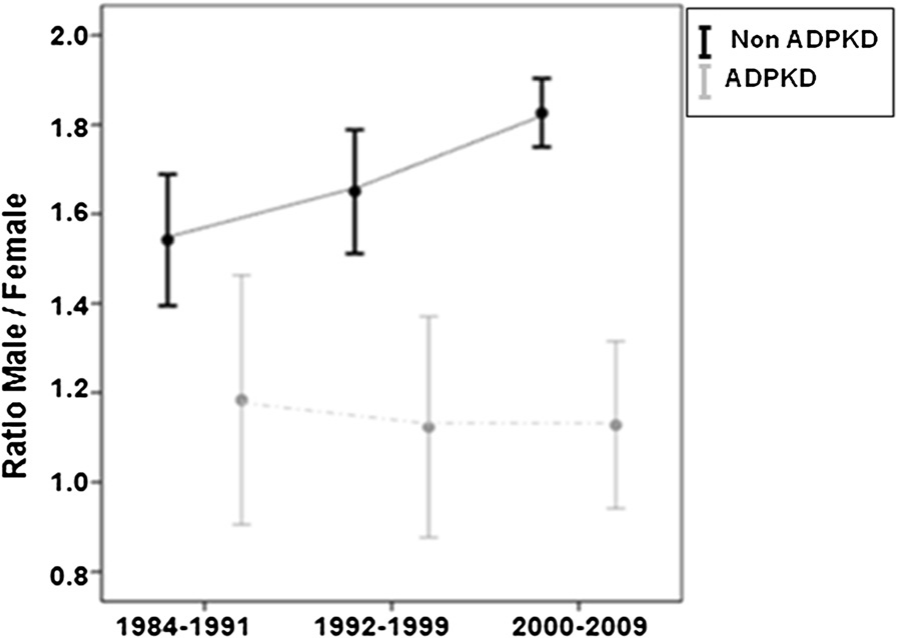
Male to female ratio in the ADPKD and non-ADPKD populations on RRT, in the three periods of the study.

Both groups contained more males than females. The ADPKD group comprised 837 (52.8%) men and 749 (47.2%) women, while the non-ADPKD group comprised 11,641 (63.1%) men and 6,806 (36.9%) women. No significant differences were identified regarding the incidence of males and females at RRT initiation in the ADPKD group (ratio, 1.1–1.2), but in the non-ADPKD group the number of men who started RRT was significantly higher than the number of women (ratio, 1.6–1.8) (Figure 2).

Table 2 shows that cardiovascular diseases, malignancies, chronic respiratory disorders and joint diseases are significantly less frequent among ADPKD patients (p < 0.001). Among the cardiovascular risk factors analysed, diabetes was significantly less frequent (p < 0.001) in ADPKD patients than in the non-ADPKD group (6.76% vs 11.89%), and no differences were observed regarding the prevalence of hypertension (86.24% vs 84.20%).

**Table 2:** Associated diseases in patients with and without ADPKD (2000–2009)

**Disease**	**ADPKD % (n)**	**Non-ADPKD % (n)**	***p***
Hypertension	86.24 (589)	84.20 (7567)	0.158
Diabetes mellitus	6.02 (42)	12.45 (1143)	<0.001
Ischaemic cardiopathy	9.42 (65)	21.66 (1954)	<0.001
Other cardiomyopathy	9.83 (68)	28.43 (2566)	<0.001
Arrhythmias	6.08 (42)	17.37 (1568)	<0.001
Cerebrovascular	8.16 (56)	13.65 (1230)	<0.001
Vascular	6.41 (44)	23.0 (2072)	<0.001
Cancer	4.93 (34)	11.36 (1026)	<0.001
Chronic respiratory	7.14 (49)	18.20 (1639)	<0.001
Tuberculosis	0.44 (3)	1.22 (110)	0.065
Liver	3.6 (25)	6.65 (600)	0.002
Gastroduodenal	7.15 (49)	10.69 (963)	0.004
Intestinal	4.82 (33)	6.35 (572)	0.110
Arthropathy	13.99 (96)	23.97 (2157)	<0.001

Most of the patients started RRT with haemodialysis in both groups. However, a prevalence analysis dated 31 December 2009 showed that transplantation was the most prevalent situation among ADPKD patients: 65.4% in the ADPKD group and 49.3% in the non-ADPKD group. The analysis also showed that fewer patients with ADPKD required erythropoietin (p < 0.001) and that they had a lower prevalence of hepatitis C virus (p < 0.001). The CRP levels were lower in the ADPKD group, but this difference did not attain statistical significance (p = 0.124) (Table 1).

Significant differences (p < 0.001) in the use of different types of vascular access at initiation of haemodialysis were also observed in both the periods analysed (1997–2003 and 2004–2009) in the ADPKD group. An increase in arteriovenous fistulae was observed (74.3% vs 78.6%), and the same was true for tunnelled catheters (2.6% vs 7.5%) and prostheses (2.6% vs 7.5%); there was also a reduction in the number of temporary catheters (22.3% vs 11.7%). When analysing patients with kidney transplants, the number of living donors increased only over the most recent years (10–11 per year), even though cadaveric transplants continue to be more frequent (95.1% of all kidney transplants).

**Figure 3:**
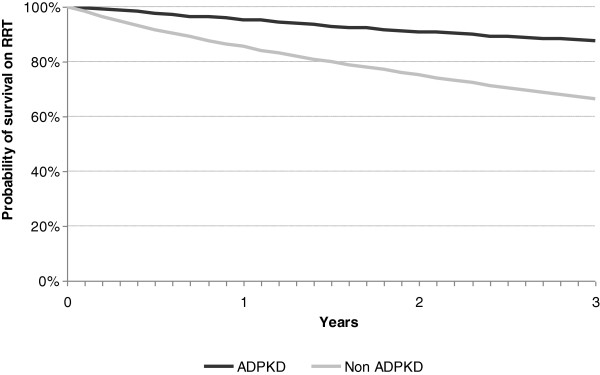
Kaplan-Meier plot of survival for ADPKD and non-ADPKD patients on RRT.

**Figure 4:**
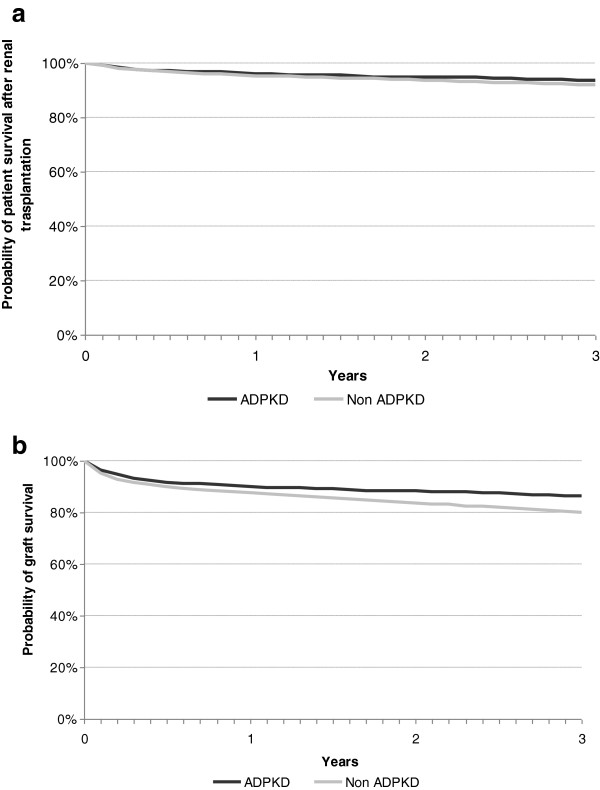
Kaplan-Meier plots of survival among transplanted patients (a) and graft survival (b) in the ADPKD and non-ADPKD populations.

The primary causes of death during the study period were cardiovascular, followed by infections, in both groups (Table 3). No significant differences were observed regarding the age of death between ADPKD and non-ADPKD patients (69.0 ± 10.0 vs 69.4 ± 12.6 years) (p = 0.41). Survival over the first 3 years of RRT was higher in the ADPKD group (p < 0.001) as well as in the subgroup of kidney-transplanted patients (p < 0.03) (Table 4; Figure 3). The renal graft survival was also longer in the ADPKD group (p < 0.001) (Table 4; Figure 4a and 4b).

**Table 3:** Causes of death of ADPKD and non-ADPKD patients after starting RRT

**Cause of death**	**ADPKD % (n)**	**Non-ADPKD % (n)**	**Age**^**1 **^**(ADPKD vs non-ADPKD)**
Cardiac	26.17 (241)	31.15 (3912)	69.1 ± 9.8 vs 68.9 ± 12.1
Vascular	18.35 (169)	16.52 (2075)	68.8 ± 10.6 vs 69.4 ± 12.6
Infection	14.44 (133)	15.57 (1955)	68.8 ± 9.6 vs 67.9 ± 13.6
Cancer	9.55 (88)	8.30 (1043)	67.5 ± 9.3 vs 67.6 ± 12.2
Hepatic	3.04 (28)	1.84 (231)	64.1 ± 2.2 vs 61.1 ± 0.9
Social^2^	3.47 (32)	6.04 (758)	75.1 ± 6.6 vs 75.7 ± 10.4
Other	12.7 (117)	9.65 (1212)	71.1 ± 9.9 vs 71.6 ± 12.4
Unknown	12.27 (113)	10.93 (1373)	67.9 ± 9.7 vs 70.0 ± 12.3

^1^No statistically significant differences when comparing the mean age at death of ADPKD patients versus Non ADPKD patients (p = 0.41).

^2^The term “social” as cause of death means: rejection of RRT by the patient or suicide.

**Table 4:** Survival rates in ADPKD and non-ADPKD patients during the first three years

	**Years**	**ADPKD (%)**	**Non ADPKD (%)**	***p***
Patients on RRT	1st	95	85	<0.001
	2nd	91	75	
	3rd	88	66	
Transplanted patients	1st	96.0	95.4	0.03
	2nd	94.8	93.6	
	3rd	93.4	91.9	
Graft survival	1st	90.0	87.4	<0.001
	2nd	88.4	83.7	
	3rd	86.2	80.1%	

## Discussion

Although there is no specific treatment for ADPKD as yet, the therapeutic advances to slow down CKD might cause one to expect that, over time, onset of ESRD in ADPKD patients will have been delayed until a later age. Our data, however, show that the age at initiation of RRT in these patients has not varied since 1984. This result is in concordance with other articles [15], but some publications [16-18] do indicate that the age at initiation of RRT in ADPKD patients has indeed increased. According to Orskov et al. [16], age at onset of ESRD among their 693 ADPKD patients, followed up for 18 years (divided into three periods), rose from 55.9 (1990–1995) to 60.6 years (2002–2007), though these findings are subject to a potential bias, given the increasing number of patients in each period and especially the rise in the number of women.

The prognosis of CKD patients who start with RRT has improved over the last few years [19,20]. In the European Registry, the percentage of patients older than 65 who started RRT increased from 22% in 1980 to 55% in 2005 [21], and this may be attributed to a better control of cardiovascular risk factors. We have not been able to find in the literature any studies comparing the age at onset of ESRD between patients with and patients without ADPKD. Based on our own results, we would stress that while, over the three periods examined, the age at initiation of RRT in non-ADPKD patients rose by more than 9 years, no such change occurred among ADPKD patients.

Epidemiological studies indicate that within the general population, the prevalence of men undergoing RRT is higher than that of women, in accordance with our findings [21]. These data are in agreement with some other publications that associate male gender with earlier initiation of RRT in ADPKD patients [22,23]. In contrast, and in accordance with other studies [16,24], we found no significant gender differences in relation to age at initiation of RRT. Our data show that male gender is a risk factor for initiation of RRT among non-ADPKD patients (ratio, 1.6-1.8), but not among ADPKD patients (ratio, 1.1-1.2).

Although most of our ADPKD patients started RRT with haemodialysis, the prevalence study revealed kidney transplantation to be the most widely used method, as expected based on the age at onset of ESRD in this population. The sparse prescription of peritoneal dialysis among ADPKD patients could be due to large kidneys, to the presence of colonic diverticula, to hernias or to massive polycystic liver disease. However, peritoneal dialysis is a first-line technique for this disease, as has been shown by several studies that revealed no differences concerning survival or risk of peritonitis [25,26]. On the contrary, it was found that ADPKD patients on peritoneal dialysis had a higher survival rate than those on haemodialysis [25].

The factors related to greater ADPKD severity are primarily carrying a *PKD1* mutation, especially if truncating, and hypertension [3,8,27]. Other relevant factors include: age at diagnosis [28,29], increased number and volume of the cysts, left ventricle hypertrophy [30], proteinuria [31], haematuria, microalbuminuria, hyperuricaemia [32], low HDL-cholesterol levels, high urinary sodium and an increase in urinary osmolality [33]. Cardiovascular risk factors increase the rate CKD progression [4,12], with both entities boosting each other, giving rise to a cardiorenal syndrome. ADPKD is a typical model of type IV cardiorenal syndrome [34], as the cause of CKD is primary renal failure and secondary cardiovascular involvement. We have observed a lower prevalence of diabetes among patients with ADPKD, but the differences in comparison with non-ADKPD patients have not been found to be significant with regard to hypertension, similarly to other studies [14,35-37]. The data published on associated diseases in ADPKD and non-ADPKD patients are either incomplete [38] or have concerned only cardiovascular events [39]. We have observed that cardiovascular diseases, malignancies and respiratory diseases are more frequent among non-ADPKD patients on RRT. This higher rate of co-morbidities in non-ADPKD patients could be explained by the higher prevalence of diabetes and by initiation of RRT at an older age. The lower cardiovascular morbidity among ADPKD patients could be associated with higher haemoglobin levels in this group [35]. In correspondence with Abbott and Agodoa’s results [35], we found that fewer ADPKD patients on dialysis need erythropoietin compared with the non-ADPKD group (77.9% vs 91.9%). CKD favours a chronic inflammation status with increased inflammatory markers such as C-reactive protein (CRP) [37]. In accordance with other articles, we observed no significant differences between the groups with regard to CRP levels, although they were lower among ADPKD patients on dialysis, probably owing to the lower prevalence of associated diseases [40].

The hugely significant genetic factor in ADPKD could be related to a fatal renal prognosis that is quite difficult to modify with the currently available therapeutic tools which improve cardiovascular factors. Stimulation of the renin-angiotensin system (RAS) has been associated with a sombre ADPKD prognosis owing to faster growth of the renal cysts [24]. The HALT trial is underway to elucidate the contribution of angiotensin-converting enzyme inhibitors (ACEIs) and angiotensin receptor blockers (ARBs) and the strict control of hypertension to the slowing of decline in renal function [41]. Increased use of RAS-blocking anti-hypertensive drugs could imply that ADPKD patients will start on RRT later and later [16]. However, although ACEIs and ARBs are widely used for ADPKD in our population (specific data not available), this assumption is not in accordance with our findings.

Kidney transplantation is for the moment the best therapeutic choice for ADPKD patients with ESRD. Longer survival of both patient and graft has been observed in recipients of kidney transplants with ADPKD [20,42], in agreement with our results. This increase in survival could be explained by the ADPKD patients’ younger age, lower rate of diabetes, lower prevalence of dyslipidaemia and anaemia, and also a lower prevalence of co-morbidities, mainly cardiovascular ones. It has not been possible to show that immunological factors may play a role in this survival difference. No explanation for a longer graft survival, other than the own patient’s survival, is available. The relatively low but increasing percentage of pre-emptive living donor transplantation is due to the transplantation policies of Spain, which has a highly successful programme for cadaveric transplantation and is encouraging living donor transplantation to overcome the current relative scarcity of cadaveric donors.

The primary cause of death among ADPKD patients on RRT, in our cohort, is cardiovascular disease, followed by infections, as reported by several other authors [43-45]. Various articles have shown higher survival of ADPKD patients on RRT as compared to non-ADPKD patients, which concurs with our data [46,47]. We found no differences as to the age of death, even though ADPKD patients started RRT earlier than non-ADPKD patients; this indicates a longer survival of ADPKD patients on RRT. Longer survival of ADPKD patients has been associated with: starting RRT at a younger age, lower prevalence of the cardiovascular risk factors and higher haemoglobin levels, all of which are factors applicable to our results [48,49]. In another study, ADPKD patients on RRT still showed a higher survival rate than diabetes-free non-ADPKD patients [50], though such a difference was not observed by other authors [51].

## Conclusion

Over the last few years initiation of RRT has been delayed in non-ADPKD patients owing to cardiovascular risk factors being better controlled. ADPKD patients have a lower prevalence of cardiovascular risk factors and also less co-morbidity, which favours longer survival. Still, over time no changes in age at onset of ESRD have occurred among ADPKD patients who have started RRT, probably because of the impact of unmodifiable genetic factors in the absence of a specific treatment. However, there is reasonable hope that disease progression in patients with ADPKD can be delayed with newer therapeutic approaches which are currently being tested.

## Competing interests

There is neither specific support nor financial disclosure for the present study. The authors declare that they have no competing interest.

## Authors’ contributions

VM and RT conceived of the study, and participated in its design and coordination. JC and EA provided data and assisted with statistics. JMD, JB, EA, JC and SM helped to draft the manuscript. All authors read and approved the final manuscript.

## Authors’ information

RT chairs the Inherited Renal Diseases Unit at Fundació Puigvert. She also chairs the working group on inherited kidney disorders within the Spanish Society of Nephrology and is the President of the Scientific Committee for the AIRG-E.

## Pre-publication history

The pre-publication history for this paper can be accessed here:

http://www.biomedcentral.com/1471-2369/14/186/prepub
